# WHEDA study: Effectiveness of occupational therapy at home for older people with dementia and their caregivers - the design of a pragmatic randomised controlled trial evaluating a Dutch programme in seven German centres

**DOI:** 10.1186/1471-2318-9-44

**Published:** 2009-10-02

**Authors:** Sebastian Voigt-Radloff, Maud Graff, Rainer Leonhart, Katrin Schornstein, Myrra Vernooij-Dassen, Marcel Olde-Rikkert, Michael Huell

**Affiliations:** 1 Department of Occupational Therapy, Centre of Geriatric Medicine and Gerontology Freiburg, University Hospital Freiburg, Germany; 2 Alzheimer Centre Nijmegen, Scientific Institute for Quality in Health Care, Rehabilitation-Occupational Therapy, Radboud University Medical Centre Nijmegen, The Netherlands; 3 University of Freiburg, Department of Social Psychology and Methodology, Germany; 4 Alzheimer Centre Nijmegen, Scientific Institute for Quality in Health Care, Radboud University Medical Centre Nijmegen, The Netherlands; 5 Alzheimer Centre Nijmegen, Department of Geriatrics, Radboud University Medical Centre Nijmegen, The Netherlands; 6 Centre of Geriatric Medicine and Gerontology Freiburg, University Hospital Freiburg, Germany

## Abstract

**Background:**

A recent Dutch mono-centre randomised controlled trial has shown that occupational therapy improves daily functioning in dementia. The aim of this present study is to compare the effects of the Dutch community occupational therapy programme with a community occupational therapy consultation on daily functioning in older people with mild or moderate dementia and their primary caregivers in a German multi-centre context.

**Methods/Design:**

A multi-centre single blind randomised controlled trial design is being used in seven health care centres (neurological, psychiatric and for older people) in urban regions. Patients are 1:1 randomised to treatment or control group. Assessors are blind to group assignment and perform measurements on both groups at baseline, directly after intervention at 6 weeks and at 16, 26 and 52 weeks follow-up. A sample of 140 community dwelling older people (aged >65 years) with mild or moderate dementia and their primary caregivers is planned. The experimental intervention consists of an evidence-based community occupational therapy programme including 10 sessions occupational therapy at home. The control intervention consists of one community occupational therapy consultation based on information material of the Alzheimer Society. Providers of both interventions are occupational therapists experienced in treatment of cognitively impaired older people and trained in both programmes. 'Community' indicates that occupational therapy intervention occurs in the person's own home. The primary outcome is patients' daily functioning assessed with the performance scale of the Interview for Deterioration in Daily Living Activities in Dementia and video tapes of daily activities rated by external raters blind to group assignment using the Perceive, Recall, Plan and Perform System of Task Analysis. Secondary outcomes are patients' and caregivers' quality of life, mood and satisfaction with treatment; the caregiver's sense of competence, caregiver's diary (medication, resource utilisation, time of informal care); and the incidence of long-term institutionalisation. Process evaluation is performed by questionnaires and focus group discussion.

**Discussion:**

The transfer from the Dutch mono-centre design to the pragmatic multi-site trial in a German context implicates several changes in design issues including differences in recruitment time, training of interventionists and active control group treatment.

The study is registered under DRKS00000053 at the German register of clinical trials, which is connected to the International Clinical Trials Registry Platform.

## Background

Dementia is a progressive, irreversible neurodegenerative disorder. The main symptoms are decline of memory and other cognitive functions associated with deterioration in daily functioning [1]. Several types of dementia are differentiated by aetiology including 72% Alzheimer, 16% vascular and 11% other types [2]. In the year 2000, there were 7.1 million people with dementia and 493 million persons of working-age [3] within Europe. Age is associated with increasing prevalence [4] with rates estimated at 6% in people aged over 65 and 30% in the over 80s [5]. Incidence rates range from 1.4% [6] to 3.2% [7]. Wancata and colleagues [3] predict the number of people with dementia in Europe to be 16 million or above in the year 2050. Bickel [8] estimates these numbers for Germany at 1.2 million in 2010, 1.7 million in 2020 and 2.3 million in 2050. In Germany, more than two thirds of people with dementia are women. 50% of community dwelling people with need of nursing care suffer from dementia. The disease constitutes the main cause of admission to long-term nursing homes with 60% of nursing home residents being affected by dementia. However, in Germany the majority of people with dementia (60%) live in the community and receive care by family members [9].

Direct costs of dementia in the year 2003 were estimated at 156 billion USD worldwide and 60.5 billion USD in the EU-25 region [10]. Direct costs in Germany amount to 5.6 billion Euros in the year 2002, including the major part of 3.6 billion Euros for dementia care in nursing homes [11]. Considering direct and indirect costs in Germany, Hallauer et al. [12] calculated dementia costs of 43,767 Euros per year and per patient, divided into 2.5% medical costs (medication, consultation, hospital stays), 29.6% nursing care costs and 67.9% costs borne by the patients' families. International studies demonstrate similar results and confirm the fact that family caregivers bear the main burden of dementia [13,14].

Deficits in daily functioning are used as threshold criteria in standardized diagnostic procedure [15,16]. Within the progress of disablement, cognitive decline leads to limitations in activities of daily living, which often affect the quality of life of patient and caregiver [17]. The new International Classification of Functioning, Disability and Health [18] is based on the bio-psycho-social model of health and consequently assumes that the negative impact of cognitive deficits on activities can be diminished by improving facilitation in the patients' physical and social environment. The task performance of people with dementia can be improved by reducing distraction and arranging clear structures in the physical environment [19]. Research on caregiver interventions provides evidence that those educational and psychosocial approaches targeting at the optimisation of the social environment delay nursing home placement [20-22]. Tailoring the activity to patients' capability may enhance activity engagement and reduce challenging behaviour [23].

A Dutch intervention programme in dementia care systematically integrated environmental adaptations, support of caregivers as well as selecting and tailoring everyday activities with regard to patients' occupational preference and cognitive abilities [24]. This programme demonstrated positive impacts on patients' daily functioning, caregivers' sense of competence in interaction with the patient, on quality of life and mood of both and on costs of dementia care [25-28].

This article presents the design of the WHEDA study evaluating, whether (1) the positive results are replicable in a German multi-centre context and (2) the results remain stable in a prolonged follow up period. The methods are reported according to the requirements and structure of the CONSORT-statement including the extensions for non-pharmacological [29,30] and pragmatic trials [31].

## Methods

### Design

In this pragmatic non-pharmacological intervention trial, a randomised controlled design is being used in seven health care centres and has the following research aims:

#### Primary outcome

To compare the effects of a community occupational therapy programme (COTP) with a community occupational therapy consultation (COTC) on daily functioning of older people with dementia.

#### Secondary outcomes

• To evaluate the effectiveness of the COTP on patients' and caregivers' quality of life, mood and satisfaction with treatment and on caregivers' sense of competence and hours of care compared to those having a COTC.

• To evaluate the cost of both interventions from a societal perspective.

The intervention group receives ten sessions of occupational therapy during five weeks of one hour duration. The control group receives one home consultation of one hour duration by an occupational therapist within the same period. Measurements for both groups are at baseline before intervention (week 0), after intervention (week 6) and at follow-up (week 16, 26 and 52). Measurements of cognitive decline are applied one week before baseline and at week 11 and week 21 after baseline.

### Participants

The seven participating centres are located throughout Germany in urban regions. The catchment areas of the centres vary from about 70,000 to 700,000 inhabitants. Five outpatient memory centres are participating and are departments of university hospitals (one neurological clinic, two psychiatric clinics and two geriatric centres). One centre is part of a municipal hospital specialising in geriatric medicine. The seventh centre, a neurological private practice, collaborates with an occupational therapy private practice. The WHEDA study centres have several years of experience in providing outpatient dementia care ranging from three to fifteen years. Their standard service comprises diagnostic work-up for dementia and related diagnoses as well as recommendation of risk reduction, dementia medication and non-pharmacological treatments. Study leaders of the centres are medical specialists (psychiatrists, neurologists or geriatricians) with six to thirteen years of experience in dementia care. The occupational therapists and assessors involved in the study have a minimum of one year's professional experience with older people or those who are cognitively impaired. The assessors' professions are physician, psychologist, occupational therapist and nurse.

The inclusion criteria are as follows:

▪ Mild to moderate dementia of the type Alzheimer's disease or mixed type diagnosed according to ICD-10 by physicians who are geriatric or geronto-psychiatric specialists with more than five years of experience. Diagnosis usually included lab investigations and CT or MRI scan of the brain.

▪ Mini Mental State Examination (MMSE [32]) ranging from 14 to 24.

▪ People dwelling at home either together with their primary caregiver or the primary caregiver provides care at least twice a week.

The exclusion criteria are:

▪ A score on the 30-items Geriatric Depression Scale is > 12.

▪ The people are in major need of nursing care (level 2 and 3. In Germany special physicians and nurses involved in allocation of sickness funds have the legal mandate to evaluate the need of nursing care according to criteria determined by law. They assign a patient to one of four levels: 0 = best, no need, 3 = worst, severe need [33]).

▪ Unstable medical conditions or severe behavioural disturbances, which do not allow participation in the study as judged by the physicians of the study centres.

Discontinuation criteria:

▪ Death of patient or primary caregiver

▪ Patient is admitted to a nursing home

▪ Development of unstable medical conditions or severe behavioural disturbances as assessed by the physicians of the study centres

### Registration and ethical considerations

The study is registered under DRKS00000053 at the German register of clinical trials, which is connected to the International Clinical Trials Registry Platform of the WHO  and thus meets the demands of the International Committee of Medical Journal Editors. The study protocol has been approved by the medical ethics committee of the University Hospital, Freiburg, Number 110/08. The study was considered as a therapeutic intervention to be carried out in subjects, whose capabilities to consent might not be possible every time. Therefore, and because the proxy caregivers are also interviewed, it was proposed that double written consent was given, ie. both from person with dementia and his/her caregiver.

### Recruitment

A leaflet with consistent and consumer-friendly information about the WHEDA study was prepared for the recruitment procedure. The recruitment period lasted nine months, from August 2008 to April 2009. Former and current patients of the participating centres with mild to moderate dementia were informed about the WHEDA study during a routine visit at the study centre or via an invitation letter. General practitioners and medical specialists who usually cooperate with the centres received information about the WHEDA study via mail, e-mail or telephone. Community nursing and dementia care services also received information letters or a telephone call. Several centres published information via the local press.

A meeting was arranged at each centre for eligible patients and their primary caregivers together in order to receive verbal and written information from the study physician about the study protocol. This included the contents of the two occupational therapy interventions, the randomisation procedures and the kind and duration of measurements. Within this, or at a further visit, the study physician received written informed consent and full agreement with the contents and procedures of the study from both patients and primary caregivers. This was essential before allowing participation in the study.

### Comparison with 'usual' dementia care

The characteristic of the study design, inclusion and exclusion criteria as well as participating centres and recruitment processes were determined in such a way, to assure that the study sample and procedures are approximately congruent with the structures and processes of usual dementia care in Germany. For this and other reasons, the design of the WHEDA study differs from the original Dutch trial. There is a higher number of participating centres and therapists; less years of experience of the therapists; as well as the inclusion of an active control treatment arm, a longer follow up period and study sites other than university centres.

### The Intervention Group (COTP)

Intervention group participants receive COTP with ten sessions of one hour duration held over five weeks. The intervention aims at the improvement of daily functioning of both the patients and their primary caregivers. The treatment of the patient focuses on enabling the successful performance of highly meaningful daily activities. Therefore, the occupational therapist explores the patient's preferences and history of activities using the one hour Occupational Performance History Interview [34]. In the second session, the occupational therapist observes the patient's ability to perform relevant daily activities and to use compensatory strategies within his/her familiar environment and then evaluates the possibility of modifying the patient's home and surroundings if necessary.

The intervention focus for the caregiver is on enhancement of successful interaction with the person with dementia by improving the caregiver's skills in communication, supervision and problem solving. In addition, the caregiver is supported in caring for his own wellbeing by enabling meaningful and recreational daily activities. Therefore, the occupational therapist explores the caregiver's preferences of activities using the one hour Ethnographic Interview [35]. He/she also observes the interaction between caregiver and patient.

After three or four sessions of interviews and observation in the familiar surroundings, the occupational therapist summarizes the meaningful activities that were named in both the interviews with the patient and the caregiver. Patient and caregiver together prioritise the proposed activities. In discussion with the occupational therapist, they identify those which are

• most meaningful and motivating for the patient (patient's perspective)

• most helpful or recreational for the caregiver (caregiver's perspective)

• most promising to be adapted and stabilised as activities that the patient and caregiver can perform successfully in the future (occupational therapist's perspective)

When one or two of the most appropriate activities are chosen, the therapist defines compensatory and environmental strategies to adapt activities and environment to the patient's habits and cognitive abilities if required. Patient and caregiver are taught to use and optimize these compensatory and environmental strategies to improve their performance of daily activities. Therefore, the caregiver is trained in effective supervision skills and receives practical and emotional support. In addition, the caregiver is taught effective problem solving and coping strategies by means of cognitive and behavioural interventions, in order to sustain the patient's and his/her own autonomy and social participation. A manual comprising a detailed description of the intervention is used [24]. These guidelines for the treatment of older people with cognitive impairments has been developed and evaluated systematically over the last ten years. This process included a literature review, theoretical modelling, an advisor panel of international experts, draft manuals, pilot testing in practice, case study analyses, pilot study and an RCT on effectiveness and on cost-effectiveness [25-28,36]. For the WHEDA study, the Dutch manual was translated into German. The main author (Maud Graff, MG) taught the content of this manual to 14 study therapists from seven centres in 16 hours of seminars. Seminar methods included presentation, videos and role play with feedback and group discussion. The study therapists had to have at least one year of professional experience in the treatment of older persons with cognitive impairments. Within the pilot phase after the seminars, every therapist had to treat at least one patient with a full treatment series of ten sessions. The therapist also had to provide a videotape of one key session (interview with patient or caregiver or shared goal finding session) and a quality report reflecting on the whole series. Videotapes were evaluated by MG and quality reports by the study manager, Sebastian Voigt-Radloff (SVR). The expenditure of time for the seminars and learning on the job within the pilot phase was about 40 hours per therapist while in the Netherlands it was 80 hours including more skill training, learning on the job and feedback.

The following arrangements were provided to enhance and evaluate the adherence of the therapists to the manual:

• A mailing list for e-mail exchange of all study therapists and the study manager.

• On-call telephone coaching by SVR for the therapists.

• Therapy evaluation form for every treatment case, where therapists assess their adherence to the manual and describe their problems within the eleven different intervention steps.

• Questionnaire, by which therapists retrospectively evaluate the whole intervention phase after it is finished.

• The study protocol demands adherence to the standard procedures of the manual. However, a therapist may deviate from the manual in cases, where strict adherence would lead to any harm to patient or caregiver; if very poor motivation; or to withdrawal. The deviation must be reported on the therapy evaluation form. In general, the manual sets standards for the procedures, but an inherent part of the treatment is the individualised tailoring of activities to patients' and to caregivers' preferences and abilities. The demands for adherence and rules for exceptions do not differ between centres, therapists or participants.

In Germany, a series of ten home visits is within the normal range of time that occupational therapists use for the treatment of older outpatients diagnosed with other diseases, such as stroke or rheumatoid arthritis. A directive governs the service delivery and utilisation of occupational therapy in German private practices. It gives permission to do home visits, but they are not often used. No additional legal or organisational structures are being added for the new intervention programme. However, home visits of occupational therapists working with people with dementia are not established yet due to the lack of evidence-based interventions in Germany. A seminar and learning time of 40 hours is quite common in the field of occupational therapy, in order to implement an innovative intervention.

### Control Group

The patients and care givers in the control group, together receive one COTC of one hour's duration by the same study therapists who carry out the treatment intervention. The visit is not time-matched to the intervention group. The control intervention is carried out as a semi-structured consultation, half is an explanation using a brochure; and half is a talk on individual problems in everyday life. Two occupational therapists with a high level of experience in dementia care prepared the brochure of ten pages especially for the WHEDA control intervention. It is based on two brochures of the German Alzheimer Society [37,38]. During a three hour session, SVR taught the WHEDA therapists the content of the control intervention, using presentations and role plays. Within the pilot phase, the study therapists of each centre have had to collect contact data of dementia care services in their region and add it to the brochure, which is then given to caregivers and patients during the home consultation. Furthermore they are obliged to carry out one full pilot COTC and write a quality report on problems and facilitation of this consultation visit. SVR evaluates this quality report. The protocol of the control intervention was determined as follows:

• 10 minute warm-up phase including the welcome and introduction of the scope and purpose of the consultation;

• 20 minute structured explanation of how patients and caregivers may deal with the consequences of dementia in every day life;

The outcome for the patient is to:

◦ stay active, undertaking usual everyday activities

◦ keep social contacts

The outcome for the care giver is to:

◦ support patient, but not to make him/her inactive;

◦ take time for self;

◦ keep social contacts and look for support for self;

◦ discuss what a successful interaction with the patient may look like, especially when challenging behaviour occurs;

◦ use data of local dementia care services such as day care clinics, caregiver or dementia outpatient groups, outpatient nursing care, home help or occupational therapy private practices.

• 25 minute talk on individual problems that arise from the patients' and the caregivers' needs;

• 5 minute completion and farewell.

The focus of the control intervention is on the encouragement to stay active in everyday life, keep social contacts and use resources within the environment.

The same arrangements as in the intervention group are provided to enhance and evaluate the therapists' adherence to the structure and procedure of the introduction to the control group. The control group protocol leaves the occasional decision to the therapist, whether the 20 minute block with structured explanations is to be timed before, after or in between, the talk on individual problems. Problems and deviations from protocol must be documented in the therapy evaluation form. Rules of procedures and exceptions apply to therapists of all study centres in the same way.

Consultations of 30 minutes up to one hour duration are common in German dementia care. However, social workers, physicians or special nurses mainly give advice on local care services, on progress and medication in dementia and on nursing care problems. The focus on occupational therapy and everyday activities is innovative, but needs no additional resources.

### Objectives

The aim of this study was to compare the effects of the COTP and the COTC on functioning in everyday life of people with mild or moderate dementia and their primary caregivers within a German routine care context. The null hypothesis is that there is no significant difference between the intervention group and the control group. The significance for rejecting this hypothesis will be tested two-sided. The secondary research questions are as follows:

• Impact of COTP and COTC on the:

◦ quality of life of patients and caregivers

◦ mood of patients and caregivers

◦ caregivers' competence in the interaction with the patients

◦ utilisation of resources for patients and caregivers

◦ time of informal care given by the primary caregivers

• Costs and feasibility of COTP and COTC

• Patients' and caregivers' acceptance and satisfaction with COTP and COTC

• Incidence of admissions to long-term nursing care homes one year after baseline

### Outcomes

The primary endpoint is the comparison between the intervention group and control group in the change in daily functioning from baseline to follow-up as indicated by the Interview for Deterioration in Daily Living Activities in Dementia (IDDD) over three post intervention time points. The WHEDA measurement scheme with dependent variables and measurement instruments is provided in table 1. The primary and most secondary outcomes are oriented to patient and caregiver. Daily functioning, quality of life, mood and daily interaction are directly related to individual everyday life and represent the user's perspective in most self- and proxy-assessments used in the WHEDA study. It is of great importance to know how long a therapeutic approach may stabilise daily functioning after the intervention is finished. The cognitive decline in dementia is a strain on patients and caregivers. Its progress varies, but major and consistent changes are mostly not observable before a half year progress of disease. Consequently, the follow-up assessments are appointed at week 16 and 26 after baseline. In order to justify a potential implementation in practice, service providers as well as political and economical decision-makers within the health care system need trustworthy evidence about an innovative intervention. Hence feasibility, acceptance and costs of active and control treatment are additional outcomes of the WHEDA study.

**Table 1:** WHEDA measurement scheme

		**Week**										
**Variable**	**Instrument**	**-1**	**0**	**6**	**11**	**16**	**21**	**26**	**32**	**38**	**45**	**52**
**Primary outcome**												
Patients' functioning in everyday life	IDDD, PRPP-Video rating		X	X		X		X				

**Secondary outcome patient**												
Quality of life	DQoL, SF-12		X	X		X		X				
Mood	CSDD		X	X		X		X				X
Resource utilisation incl. informal care	Adapted RUD (+ diary as reminder)		X	X	X	X	X	X	X	X	X	X
Satisfaction with treatment	5-point-Likert-scale and comments			X								
Long-term institutionalisation	Adapted RUD (+ diary as reminder)		X	X	X	X	X	X	X	X	X	X

**Secondary outcome caregiver**												
Competence in interaction with patient	SCQ		X	X		X		X				X
Quality of life	DQoL, SF-12		X	X		X		X				X
Mood	CES-D		X	X		X		X				X
Resource utilisation	Adapted RUD (+ diary as reminder)		X	X	X	X	X	X	X	X	X	X
Satisfaction with treatment	5-point-Likert-scale and comments			X								

**Control measures**												
Demographic data	Socio-demographic questionnaire		X									
Depression	GDS	X										
Cognition	MMSE	X			X		X					
Medication	Adapted RUD (+ diary as reminder)		X	X	X	X	X	X	X	X	X	X
Periods of illness	Adapted RUD (+ diary as reminder)		X	X	X	X	X	X	X	X	X	X

**Process measures**												
Adherence to manual (therapist)	Evaluation form for every treatment			X								
Adherence of patient and caregiver	Structured therapist questionnaire				When treatment period is finished							
Adherence to protocol (therapist)	Structured therapist questionnaire				When treatment period is finished							
Adherence to protocol (study centres)	Deviations of protocol schedule	Continuous registration										

IDDD = Interview of Deterioration in Daily Living Activities in Dementia

PRPP = Perceive, Recall, Plan and Perform System of Task Analysis

DQoL = Dementia Quality of Life Instrument

SF-12 = Short Form of the SF-36 measuring health-status

CSDD = Cornell Scale for Depression in Dementia

RUD = Resource Utilization in Dementia

SCQ = Sense of Competence Questionnaire

CES-D = Center for Epidemiologic Depression Scale

GDS = Geriatric Depression Scale

MMSE = Mini Mental State Examination

The quality of the measurements is enhanced by an extended check and report of metric properties and via following methods of process improvements:

• An 8 hour seminar trains the study assessors in interviewing patients, taking videotapes of patients' everyday activities, handing over questionnaires to caregivers and checking completed forms for completeness and plausibility.

• The main assessor of every study centre performs two pilot assessments including videotaping and his/her deputy performs one assessment.

• All questionnaires and video tapes of pilot assessments and, additionally, a quality report reflecting problems and facilitation within this pilot phase, are sent to the WHEDA-headquarters. Video tapes are evaluated by a rating specialist for the Perceive, Recall, Plan and Perform System of Task Analysis (PRPP) and questionnaires and quality reports by SVR. A positive rating of all pilot material is required, in order to achieve approval for starting the study.

• The 'blind' raters of the videos give feedback on visibility and appropriateness of instructions on every tape. Based on this feedback, the WHEDA headquarters gives assessors recommendations for the improvement of videotaping in case the visibility or instructions are lower than the defined quality level.

• A mailing list for an online exchange of study assessors has been established.

• Any problem with assessment arising during the study is added to the problem solving list. Problems are promptly analysed and instructions for problem solving are given by SVR via this list, which is sent out to the study centres as soon as a new item is added.

#### Primary outcome measures

• The Interview of Deterioration in Daily Living Activities in Dementia (IDDD [39]) consists of two scales recording the caregivers' rating of patients' initiative and performance of daily living activities. The *Initiative Scale *measures the initiative for (1) washing oneself, (2) making tea or coffee, (3) dressing, (4) combing one's hair and brushing one's teeth, (5) shopping, (6) using the phone, (7) preparing a meal, (8) cleaning the house or doing minor repair work and (9) handling finances. The *Performance Scale *records the need of assistance in the performance of the same nine activities plus eating and using the toilet. Both domains of the IDDD are constructed as five-point-Likert-scales with the ratings never = 4, seldom = 3, sometimes = 2, often = 1, always = 0 in the *Initiative Scale *and values vice versa in the *Performance Scale*. Scores of the *Initiative Scale *range from 0 to 36 (higher scores indicate less initiative). The *Performance Score *ranges from 0 to 44 (higher scores indicate higher need for assistance). In a Spanish sample of 451 persons, the IDDD demonstrated great internal consistency (Cronbach's alpha = .99), reproducibility (intraclass correlation coefficient ICC = .94) and significant differences between groups of patients with mild cognitive impairment and patients with dementia [40]. In a Dutch sample of 25 primary and secondary caregiver pairs, the IDDD interrater reliability was high (ICC: .85 for the *Initiative Scale *and .74 for the *Performance Scale*, [39]). Within the Dutch original study the *Performance Scale *demonstrated a very high responsiveness by indicating clinically relevant improvement in 78% of cases in the intervention group and 12% of cases in the control group 6 weeks after baseline and 82% and 10% respective after 12 weeks [26].

• With the Perceive, Recall, Plan and Perform System of Task Analysis (PRPP) occupational therapists evaluate a usual daily activity as performed by the patient as well as the impact of the cognitive performance component on this performance [41]. In the first of two assessment stages, the rater defines single steps of the performed activity to be analysed and identifies any activity step in which errors of accuracy, omission, repetition or timing occur. The number of activity steps rated as incorrectly performed is divided by the total number of activity steps, resulting in an independence-score indicated in percent (100% = all steps are error-free). In the second stage, the therapist rates 34 items of observable behaviour representing the patients' use of information processing strategies while performing the activity. On the three-point-Likert-scale the value 1 indicates incomplete or non-timely or jeopardising task performance or the need of significant assistance. Value 2 indicates a complete and non-jeopardising task performance but with problems in timing and possible need of assistance. Value 3 indicates a complete, timely and non-jeopardising task performance without any need of assistance. The 34 unweighted items are summed up by four main scales Perceive, Recall, Plan and Perform each with three subscales and a PRPP global processing score ranging from 35 to 103 (higher scores denotes better performance). The PRPP-System was systematically developed over the last 12 years including the following steps and results [41]:

◦ Content validity was shown in a comparative analysis of the same activities of daily living in a sample of 25 healthy adults and a sample of 20 persons with brain injury. Variations of individual performance but no errors occurred within the healthy sample whereas clearly observable errors within the sample with brain injury could be catalogued in the four main categories accuracy, omission, repetition or timing. Interrater agreement of six raters was high (>90%) for both samples.

◦ A microanalysis of more than 4000 errors occurring in the performance of dressing, eating and grooming of 45 patients with brain injury revealed four broad errors types in cognitive processes of perceiving, recalling, planning and performing. Error analyses in subsequent investigations of other samples led to a stable pattern of 34 items which describe observable behaviour.

◦ Aubin et al. [42] found moderate indicators for the construct validity of the PRPP in a small sample of 10 adults with schizophrenia. The scores of the *PRPP total *and three of four scales differed significantly comparing a simple and a complex activity of meal preparing (p ≤ .01). The correlation between PRPP global score on the complex task and the Independent Living Skills Survey [43] was moderate (*r *= .67, p = .03).

◦ Interrater reliability of three raters in a sample of 15 adults with schizophrenia was moderate (ICC: *PRPP total *.77, *Perceive *.65, *Recall *.65, *Plan *.69, *Perform *.63 [42]). An investigation of interrater reliability of nine raters in a sample of five adults with brain injury and symptoms of agitation and confusion demonstrated similar results (ICC: *PRPP total *.60, *Perceive *.59, *Recall *.59, *Plan *.51, *Perform *.53 [44]. Nott and colleagues (2008) found fair test-retest reliability and high internal consistency (ICC > .8).

◦ A high responsiveness of the instrument was indicated by significant improvement and large treatment effects as measured by the PRPP in seven adults with post-traumatic amnesia and symptoms of agitation [45].

Within the WHEDA-study, videotapes of two activities familiar to the patient and chosen out of a list of 28 defined activities of daily living are scored with the PRPP. Less and more difficult variations are described for every activity and can be applied according to the patient's cognitive ability. The WHEDA assessors must document the variant, which is noted at the first measurement time point and initiate and tape this variant at the follow up measurements.

#### Secondary outcome measure

• The Dementia Quality of Life Instrument (DQoL) is a 30-item self-assessment. 5 subscales and one global item are assessed with a five-point-Likert-scale: *positive affect *(6 items), *negative affect *(11 items), *feelings of belonging *(3 items), *self-esteem *(4 items), and *sense of aesthetics *(5 items). The ranges of scores differ from scale to scale (from 5-15 to 5-55). Higher scores indicate better quality of life, except in the subscale *negative affect*. The DQoL was developed specifically to be completed by people with dementia. The development process included consultation with people with dementia, caregivers, and professional care providers. In an American sample, 96% of 99 people with dementia (MMSE > = 13) were able to respond to questions appropriately [46]. This study revealed moderate to high internal consistency (.67 - .89) and a good two-week test-retest reliability (.64 - .90). There were no significant differences between patient groups with mild (MMSE > 17) and moderate (MMSE < = 17) dementia severity in terms of scale reliability. Correlation of DQol subscales and the Geriatric Depression Scale ranged from -.42 to -.64. A Japanese study replicated similar results for understanding, internal consistency and convergent validity at baseline and in a one year follow up. The sample consisted of 72 people with dementia with MMSE score > = 13 [47,48]. In a British study on longitudinal change in quality of life, 60% of people with a MMSE of 10 could complete the DQoL [49]. In an American study with 67 patients diagnosed with mild cognitive impairment or mild Alzheimer's disease, a three-factor solution emerged reliably from factor analyses corresponding to positive affect, negative affect and aesthetics. This DQoL factor structure was nearly identical between ratings of patient and caregiver [50]. Within the WHEDA study, the DQoL is applied to primary caregivers as self-assessment. With the patients, it is performed as an interview, in order to ensure the validity of rating. Patients are asked to answer three screening questions and data will not be collected, if more than one answer is missing.

• The SF-12 is the reduced version of the SF-36, a generic self-assessment evaluating health status and disability. The instrument generates a mental and a physical component summary score from 12 weighted items with response categories varying from dichotomous, to six-point scales using a complex algorithm based on norm data of a major American sample. Higher scores indicate better health status. Metric properties are excellent [51] and German norm data are available [52]. The instrument supports the calculation of Quality Adjusted Life Years (QALY). The SF-12 has been applied to people with dementia recently [53] and to caregivers [54]. The SF-12 appears to identify under-reported mental health problems in dementia caregivers [54]. In addition, this assessment tool seems to be appropriate for the evaluation of health-related quality of life from both the caregiver's and the patient's perspective. Arlt and colleagues [53] found that family caregivers tend to rate the patient's health-related quality of life lower than the person with dementia him/herself. For that reason the SF-12 is applied in the WHEDA-study to both the caregiver and the patient. In order to ensure quality of patients' data, the WHEDA-assessor applies the SF-12 as an interview to the patient and checks his/her understanding by means of the DQoL screening questions.

• In the Sense of Competence Questionnaire (SCQ [55]), caregivers rate their agreement with 27 statements on the experience of caregiving on a five-point-Likert-scale (from *agree very strongly *to *disagree very strongly*). The SCQ records three domains: *Consequences of involvement in care for the personal life of the caregiver *(8 items), *satisfaction with one's own performance as a caregiver *(12 items) and *satisfaction with the person with dementia as a recipient of care *(7 items). Scores range from 27 to 135 (higher scores denote a greater sense of competence). The three domains of the SCQ and its internal consistency are confirmed in validation studies involving people with stroke and dementia (variance explained by three factors: 49% respective 42%, Cronbach's alpha: .68 - .77, .83 - .85 respectively [56,57].

• The Center for Epidemiologic Depression Scale (CES-D) [58] is frequently used to evaluate mood and depression levels of persons caring for patients with dementia [27,59-62]. Caregivers rate the occurrence of 20 symptoms on a four-point Likert Scale (from *seldom *to *mostly*). Scores range from 0-60, lower scores indicate less depressive indicators. Robust metric properties of the CES-D are established [63]. Correlations with Hamilton Depression Scale and Becks Depression Inventory are moderate to high (.49 to .94). Sensitivity to change in individuals within samples treated with anti-depressive drugs was significant (.0001 to .0004). Cronbach's alpha of .89 indicates high internal consistency. In the WHEDA study, the CES-D is applied as self-assessment.

• With the Cornell Scale for Depression in Dementia (CSDD), the caregiver rates 19 symptoms of the patient within four domains: (1) *mood and related signs *(2) *behavioural disturbance *(3) *cyclic function and ideational disturbance *and (4) *physical signs *[64]. All 19 symptoms are rated on a three-point continuum: 'absent', 'mild or intermittent' or 'severe'. Scores range from 0-38, lower scores indicate less depressive characteristics, nine or more points indicate a depressive disorder. Müller-Thompsen et al. [65] found significant high correlations between the CSDD and three other scales for depression in a sample of patients with mild Alzheimer Disease (Geriatric Depression Scale: .70; Montgomery and Åsperg Depression Scale: .93; Nurses Observation Scale for Geriatric Patients, Mood: .72) and high correlations with the Montgomery and Åsperg Depression Scale (.74) in the more severely impaired group. Cronbach's alpha of .81 and .82 indicated high internal consistency in both, the mild and severe dementia group. The validation study of Korner and colleagues [66] revealed high inter-rater reliability for a sub-sample of 15 people with dementia videotaped and rated by independent raters (ICC: .84). Lam et al. [67] compared four depression scales in 88 elderly outpatients with dementia. They found different cut-off values for sensitivity and specificity of the CSDD in subgroups of patients with mild (91.7%, 80% respectively, cut off 6/7) and moderate to severe dementia (70%, 87% respectively, cut off 12/13).

• The primary caregiver provides data on his/her own and the patient's resource utilization in a self- and proxy-report (adapted Resource Utilization in Dementia RUD [68]). The adapted RUD records resource utilization in the following fields:

 a. Medical care for the patient: (1) periods of illness, nights in hospital or nursing home, number of consultations and purchase of technical aids within the last six weeks. (2) Number and duration of therapy sessions (such as physical or psychotherapy), nursing care or home care and visits to day care groups within the last two weeks;

 b. Informal care for the patient: (1) Hours per day of the primary caregiver for assistance in activities of daily living, instrumental activities of daily living and supervision; and also hours per day the patient is without supervision. (2) Hours of supervision by others, informal or professional caregivers within the last two weeks (as recommended by Neubauer et al. [69]);

 c. Medication of the patient within the last two weeks;

 d. Medical care for the caregiver: (1) periods of illness, nights in hospital or nursing home, number of consultations and purchase of technical aids within the last six weeks. (2) Number and duration of therapy sessions, nursing care or home care, visits to groups for caregivers of people with dementia within the last two weeks;

 e. Amount of work per week and days absence from work of the primary caregiver;

 f. Medication taken by the primary caregiver within the last two weeks.

Wimo and Nordberg [70] found fair to high correlations between times of caregiving as observed by a special team and estimated in RUD by the caregivers in twenty institutionalized persons with dementia (correlation coefficients in ADL-time .81, *p *< .001; in IADL .29, *p *= .03; supervision .51, *p *< .001). In the WHEDA-study, the adapted RUD is applied at every measurement time point after recruitment, in order to record the full resource utilization until the one year follow up. For supporting the retrospective data collection, the caregiver keeps a diary of periods of illness, nights in hospital or nursing home, number of consultations and purchase of technical aids.

• Patient and caregiver rate their satisfaction with experimental and control treatment on a five-point Likert scale on five items: How (1) patient, (2) sympathetic and (3) encouraging was the therapist? How much did the treatment deal (4) with your everyday life and (5) goals that are important to you? How satisfied are you in general with the treatment? The questionnaire also asks for comments and suggestions.

#### Control measures

• The Sociodemographic Questionnaire provides data on the patient's and primary caregiver's age, sex, socio-economic and educational status, relation between patient and caregiver and their housing. It is a self- and proxy-report of the primary caregiver.

• The study physician evaluates the patient's cognitive status using the Mini Mental State Examination, a broadly applied performance test with established metric properties [32]. The MMSE ranges from 0 to 30. The higher the score the better the cognitive status.

• The Geriatric Depression Scale (GDS [71]) is a self-rating scale consisting of 30 questions to be answered dichotomously as 'yes' and 'no'. The GDS score ranges from 0 to 30; higher scores indicate major depressive indicators. The cut-off point for depression is 12. Correlation of GDS and CSDD scores was .70 in a German sample of 140 patients with mild dementia [65]. The authors found sufficient internal consistency in subgroups of patients with mild (MMSE ≥ 18) and moderate to severe (MMSE<18) dementia (Cronbach's alpha: .83, .74 respectively).

#### Process measures

• Problems during treatment and adherence to the manuals are recorded by an evaluation form for every treatment case in both study groups.

• The study therapists evaluate their adherence to the study protocol as well as barriers and facilitators of the treatment process within a self-report questionnaire for the relevant period (seminar, pilot phase, treatment series). In addition, this questionnaire collects the therapists' ratings and comments on the adherence of patients and caregivers to therapeutic recommendations (interventions group only, since follow up visits are missing in control group and control for adherence is not possible). The summarised self-report results are reflected in a focus group discussion for all therapists involved in the study.

• Further indicators of adherence to protocol are the numbers of treatments, assessments and medical control visits inside, versus outside, the protocol schedule. Additionally, the completeness of measurements at every time point is evaluated (eg. videotape or patient interview might be missing due to reduced cognitive capacity).

### Sample size

**Figure 1:**
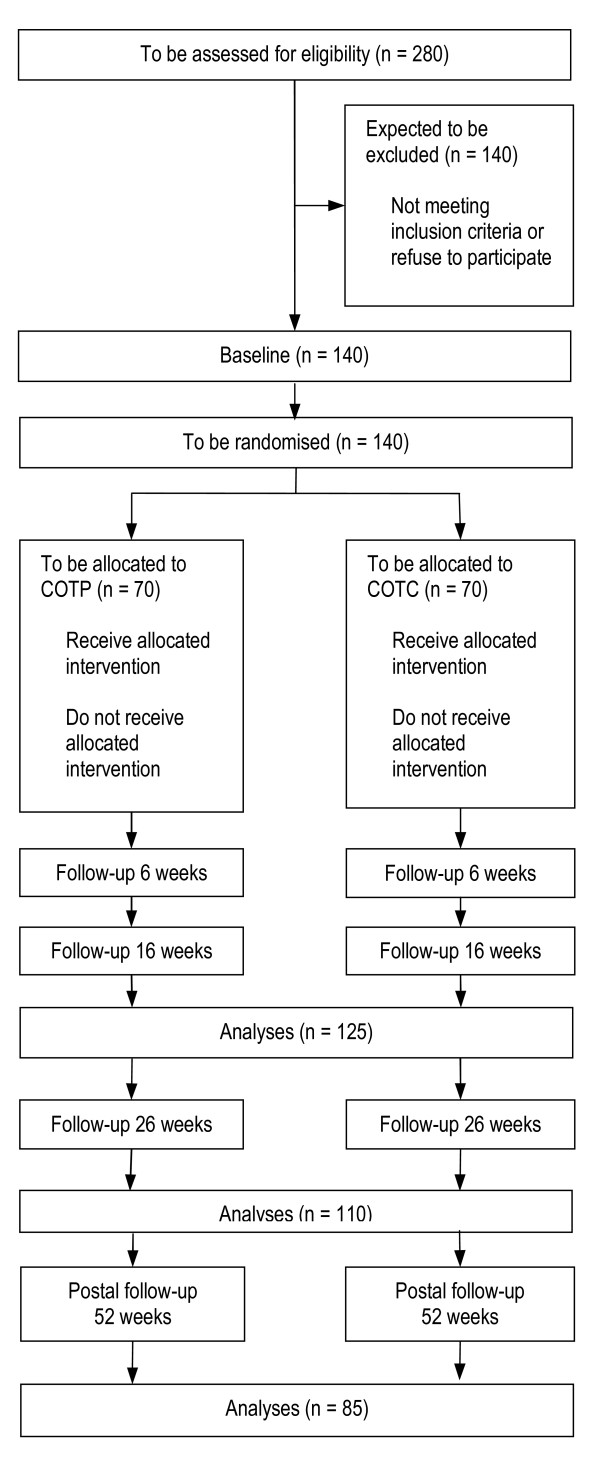
**WHEDA flow chart (planned progress)**.

Although the Dutch original randomised control trial [26] found very large effect sizes on the performance scale of the Interview of Deterioration in Daily Living Activities in Dementia (at 12 weeks follow-up d-value = 2.4), the power of the WHEDA-study is calculated conservatively in order to detect small effects. The rationale is the change in design; that there is an active control treatment, less professional experience in the experimental treatment in Germany, less patients per therapist and a longer follow-up in a sample with expected progress in dementia, may reduce the expected effects. The power calculation is based on an analysis of variance of two groups and four measurement time points (since in the postal follow up at 52 weeks the IDDD is recorded, too). Hypothesising an alpha of .05 (two-sided), a power of .80 and a correlation of .7 between four measurement time points, a sample of 84 persons is needed, in order to detect an effect of f = .10 [72]. According to drop out rates of the Dutch study, 280 patients must be screened and 140 recruited (see figure 1).

### Randomisation

After the recruitment of an individual, the physician of the study centre e-mailed the request of randomisation to a statistician at a distant site. The physician also informed the assessor (and optionally a co-ordinator of the study centre) and asked him/her to schedule all assessment dates according to the protocol. After the referral by the centre physician, the independent statistician allocated the individual to COTP or COTC. The randomisation sequence was computer-generated with blocking by centre and groups of two persons, without stratification and in a ratio of 1:1. Group allocation was e-mailed to the therapists at the study centre. The therapists decided who would be responsible for this case and the lead therapist confirmed reception of group allocation to the independent statistician. In addition, the lead therapist informed his centre team that he had received group allocation. In order to avoid any contamination, the centre therapists must schedule treatment sessions on their own. He/she also has to fax the records to the WHEDA-headquarters and retain their records, strictly separated from any other centre staff. Masking of patients and caregivers is not possible due to the different quantity of home visits between intervention and control group. Assessors might be contaminated by hints of patients or caregivers during the assessments at home. Assessors, patients and caregivers are asked to avoid any talks about the treatment. The extent of assessor 'blinding' will be evaluated by the comparison of actual group assignment and the assessor's estimation of group assignment. The procedure of video-rating ensures the full 'blinding' of the Dutch raters of an individual's assignment to group. Independent staff of the WHEDA-headquarters check and 'clean' the videotapes from any hint of group assignment or personal data, before the tapes are transferred to the Netherlands.

### Statistical methods

Data are entered via special MS Access entry masks automatically controlling for data plausibility. In addition, sections of entered data are checked for typing errors by hand, in order to ensure an error rate lower than 0.2%. Baseline measures and demographics of participants significantly differing at baseline in intervention and control group and control measures are used as co-variates in an analysis of variance with repeated measures with two groups and four measurement time points. If the diagnosis of extent and type of missing data reveal that the precondition is given, the Full Information Maximum Likelihood (FIML) method will be applied for data replacement [73]. The primary intention-to-treat analysis, including all participants with valid data (whether they did or did not receive the intervention), will be compared with a per protocol analysis excluding all participants with documented deviations from the protocol (ie. not receiving the entire intervention or receiving an incomplete follow-up). The comparison of these groups will be adjusted for baseline imbalance and process variations. Variations in the adherence to protocol; to the treatment manual and to therapeutic recommendations; variations in medication and progress of cognitive decline as well as variations in the quality of supporting or hindering structures and processes at the study sites are considered as possible mediators. All statistical tests will be two-sided on an alpha level of .05.

## Discussion

The WHEDA-study evaluates whether the improved effects on daily functioning in people with dementia, found in a Dutch RCT within a community occupational therapy programme, can be replicated in a single blind German randomised controlled multi-centre design with prolonged follow-up assessment. The transfer from the Dutch mono-centre design to the pragmatic multi-site trial in a German context implicated several determinations in design issues.

 1. The decision for a multi-centre design is justified by a short time frame for recruitment and the necessity to anticipate the variability of contexts possibly given later when the intervention might be delivered in routine care.

 2. An active control intervention is applied, since a consultation on how to deal with the consequences of the disease is normal in outpatient dementia care in Germany and the WHEDA-study aims at the evaluation of potential effects additional to standard care. Furthermore waiting group control conditions may lead to disappointment and feelings of neglect in the waiting group after randomisation and may enhance the contrast between intervention and waiting group.

 3. The follow-up period is prolonged from 12 weeks in the Dutch trial to 26 weeks in the WHEDA study, in order to assess the long-term treatment potential to reduce the negative consequences of cognitive decline in everyday functioning. The additional one-year follow-up intends to evaluate the impact of interventions on the incidence of nursing home placements.

 4. The Assessment of Motor and Process Skills (AMPS) used in the original Dutch study was replaced by the PRPP, since AMPS was not available in Germany and the PRPP shows better feasibility. Since PRPP pilot data are not available, the power calculation was based on the IDDD.

 5. The 14 German occupational therapists with a minimum of one year's experience in the treatment of people who are cognitively impaired, undertook a 16 hours post-graduate skills training course and exercise their skills in training on the job for about 35 hours within the WHEDA pilot phase. The two Dutch occupational therapists had four years experience in the treatment of people who were cognitively impaired and each had 240 hours of experience in treatment according to the guidelines after post-graduate skill training course of 80 hours. The WHEDA recruitment period of nine months is shorter than in the Dutch trial which had three years and nine months recruitment time. The reduced recruitment period, the extended number of involved therapists and the shortened seminar time all match routine conditions, which would be found in an implementation of standard dementia care.

 6. The WHEDA study does not determine stable medication for people with dementia as inclusion criteria, since changes in medication are common in dementia care. In contrast to the Dutch original trial, the WHEDA study is kept open to patients, for whom occupational therapy goals cannot be defined before the start of the treatment. This is congruent to the routine situation, since German physicians cannot explore possible occupational therapy goals before prescribing this treatment.

According to the arguments listed above, effects as revealed by the Dutch original study may be reduced in the multi-centre prolonged WHEDA design. For this reason, the statistical power was calculated conservatively and a process evaluation assessing variations in settings and delivery processes as well as treatment fidelity and adherence to therapeutic recommendations was planned. Adjusted analyses considering the variations in settings, structures and processes should be able to provide explanations for outcomes differing between the Dutch original study and the German replication.

## Competing interests

The authors declare that they have no competing interests.

## Authors' contributions

MG, MH, RL and SVR contributed to study conception and design. SVR drafted and MG helped to draft the manuscript. MH, MOR and MVD revised the manuscript critically for important intellectual content. KS and SVR participated in data and study management. KS and RL performed the statistical analysis. All authors read and approved the final manuscript.

## Pre-publication history

The pre-publication history for this paper can be accessed here:


